# Evaluation of muscle activity, bite force and salivary cortisol in children with bruxism before and after low level laser applied to acupoints: study protocol for a randomised controlled trial

**DOI:** 10.1186/s12906-017-1905-y

**Published:** 2017-08-08

**Authors:** Mônica da Consolação Canuto Salgueiro, Carolina Carvalho Bortoletto, Anna Carolina RattoTempestini Horliana, Ana Carolina Costa Mota, Lara Jansiski Motta, Pamella de Barros Motta, Raquel Agnelli MesquitaFerrari, Kristianne Porta Santos Fernandes, Sandra Kalil Bussadori

**Affiliations:** 1 Postgraduate program in Biophotonics Applied to Health Sciences, Nove de Julho University, Rua Vergueiro, 249 - Liberdade, São Paulo, SP CEP 0154001 Brazil; 2 Postgraduate program in Rehabilitation Sciences, Nove de Julho University, São Paulo, Brazil; 3 Management in Health Systems, Nove de Julho University, São Paulo, Brazil; 4 Dentist Surgeon, APCD, São Paulo, Brazil

**Keywords:** Bruxism, Child, Occlusal splints, Bite force, Acupuncture, Laser, Muscle activity, Salivary cortisol

## Abstract

**Background:**

Bruxism is a repetitive activity that causes tooth wear, audible sounds, and discomfort. Preventive measures have been studied for conditions that can exert a negative influence on physiological development in children. Low-level laser therapy administered over acupoints is an effective, painless, low-cost treatment option that has achieved good results. Thus, the aim of the proposed study is to evaluate changes in muscle activity, bite force and salivary cortisol in children with bruxism after the application of low-level laser to accupoints.

**Methods:**

The children will be randomly allocated to four groups of 19 individuals: G1 - low-level laser; G2 - occlusal splint; G3 - placebo laser; and G4 - control (without bruxism). The BTS TMJOINT electromyography will be used to determine muscle activity and a digital gnathodynamometer will be used to measure bite force. Salivary cortisol will be analysed at baseline as well as one and six months after treatment. Two-way ANOVA will be employed and complemented by Tukey’s test.

**Discussion:**

Bruxism is a repetitive activity of the masticatory muscles that can have negative consequences if not treated, such as tooth wear, noises, discomfort and anxiety. Thus, control and treatment measures should be taken. Although low-level laser therapy over acupoints has been indicated for children, the effects of this treatment modality have not yet been studied.

**Trial registration:**

NCT02757261 on 8 April 2016. This study protocol received a grant from the Brazilian fostering agency São Paulo Research Foundation (FAPESP: #2015/24731-0).

**Electronic supplementary material:**

The online version of this article (doi:10.1186/s12906-017-1905-y) contains supplementary material, which is available to authorized users.

## Background

Bruxism is a repetitive activity of the masticatory muscles characterised by grinding and/or clenching one’s teeth or movements of the mandible [1–3]. This condition is classified as either primary or secondary [1]. With primary bruxism, there is no evident medical, systemic or psychiatric cause, whereas secondary bruxism is associated with a clinical, neurological or psychiatric disorder, iatrogenic factors or sleep disorder [1, 4]. Awake (or diurnal) bruxism is characterised by clenching one’s teeth during waking hours and sleep bruxism is the unconscious activity of grinding or clenching one’s teeth during sleep with the production of audible sounds [1, 4–6].

Divergent opinions are found on the aetiology of sleep bruxism. Some authors state that central disturbances are the main cause. In this theory, muscle hyperactivity is caused by instability in the basal ganglia; synapses change the way that they function, altering between inhibitory and excitatory neurons [7]. Another theory assumes that malocclusion is the primary cause of grinding and clenching the teeth, as occlusal maladjustment reduces masticatory muscle tone; with occlusal imbalance, the activity of motor neurons of the masticatory muscles could be initiated by periodontal receptors [7]. Sleep bruxism can be measured using an index score (e.g., rhythmic masticatory muscle activity [RMMA]). Such indices measure the number of bruxism episodes per hour of sleep. The Tooth Wear Index quantifies occlusal and incisal wear to determine its severity and prevalence, with a particular cut-off point considered indicative of sleep bruxism [8]. There is also the theory that orofacial pain stems from a lack of adequate rest time between muscle activities, which leads to muscle overload and pain; however, some authors have failed to prove this [9]. Bruxism is also widely associated with stress and anxiety [10–13].

The frequency of bruxism is high in childhood, with prevalence rates ranging from 13.5 to 33% [14, 15] and this condition can cause harm to the stomatognathic system. Childhood bruxism is difficult to diagnosis and ideal treatment has not yet been established. Physical therapy is currently used to treat this condition and the most commonly employed methods are transcutaneous neuromuscular stimulation, microcurrent electrical neuromuscular stimulation, cryotherapy, ultrasound, infrared therapy, kinesiotherapy, massage therapy, acupuncture, low-level laser therapy (LLLT) and occusal splint usage [16, 17]. According to Solberg et al. [18], an occlusal splint reduces muscle activity and provides greater patient comfort. Occlusal splint usage seems to reduce tooth grinding, masticatory muscle activity and orofacial pain [19]. Although occlusal splints are widely employed for the treatment of bruxism, no specific strategy has been established for the cure of this condition. Thus, further studies are needed [18, 20].

LLLT is a non-invasive, low-cost treatment. The irradiation of trigger points constitutes effective treatment for orofacial pain as well as the reduction of swelling and hyperemia [21]. Acupuncture has also been successfully used for the treatment of bruxism, achieving a reduction in the activity of the masseter and anterior temporal muscles as well as a reduction of anxiety [22]. The stimulation of particular acupoints can alter blood circulation dynamics and promote muscle relaxation, thereby alleviating muscle spasms, inflammation and pain. Moreover, such stimulation leads to the release of hormones, such as cortisol and endorphins, thereby promoting an analgesic effect [23]. The stimulation of acupoints can be achieved with the use of needles, infrared irradiation, electrical current or laser [24]. The latter method is indicated for children because it is painless and has a shorter exposure time per acupoint [25–27]. However, the use of LLLT over acupoints has not yet been tested on children with bruxism. Therefore, the aim of the proposed study is to investigate changes in maximum bite force (measured using a gnatodynamometer) one and six months after low-level laser therapy over acupoints and occlusal splint usage compared to muscle hyperactivity at baseline in children. The secondary outcomes are the measure of pain (VAS scale), muscle activity (using the BTS TMJOINT® electromyograph) and salivary cortisol.

## Methods

This is a protocol for a randomised, controlled, clinical trial. The project received approval from the Institutional Review Board of Nove de Julho University (Brazil) under process number 1.333.636. The participants and legal guardians will receive clarifications regarding the objectives and procedures and will sign a statement of informed consent agreeing to participate in the study.

### Sample size calculation

For the calculation of the sample size, the researcher will specify that an increase in the standard deviation of the responses for which the null hypothesis will be rejected is *P* = 20%. Adopting a maximum significance level of α = 0.05 and a minimum test power of 80%, the number of subjects per group will be *n* = 15.75, which will be increased by 20% to compensate for possible dropouts, leading to 19 subjects per group (total: 19 × 4 = 76 subjects).

### Exclusion criteria

Individuals that use muscle relaxants, those with temporomandibular disorder, cerebral palsy, physical or psychiatric disorders (i.e., anxiety, persistent delusional disorder, acute and transient psychotic disorders, schizoaffective disorders and mood disorders) and those currently undergoing another treatment for bruxism will be excluded from the study.

### Inclusion criteria

Male and female children with sleep bruxism aged six to eight years with no physical or psychiatric limitations that may compromise the proposed therapies will be included. All individuals will need to have the first molars in Angle Class I and be free of dental caries. A clinical examination of tooth wear and the reports of parents/caregivers regarding tooth clenching/grinding will be used for the diagnosis of bruxism, following the criteria established by the American Academy of Sleep Medicine [1]. A questionnaire adapted from Serra Negra [28] and Manfredini et al. [29] will also be used to assist in the diagnosis of bruxism based on these two signs and symptoms (dental wear and parent’s report of tooth clenching or grinding) (Additional file 1).

### Randomisation and interventions

Randomisation will be performed using Microsoft Excel (version 2013). Seventy-six patients will be randomly allocated to the four groups. Randomisation will be in block form (groups of four patients).

Seventy-six opaque envelopes will be identified with sequential numbers (1 to 76) and each will contain information regarding the corresponding group following the established random order. The envelopes will be sealed until the time of treatment.

Patients will be allocated as follows:

Group 1 will receive low-level laser over acupoints; Group 2 will use an occlusal splint; Group 3 will receive placebo laser therapy over acupoints; and Group 4 (control group) will be composed of children without bruxism and will not receive any type of treatment. Parents/guardians will received guidance throughout the study. In G1, low-level laser (power: 70 mW; energy density: 1.675 mW/cm^2^; 12 points irradiated; 1 J per point for 20 s; 12 sessions; 12 J/session; twice a week) will be applied directly to the skin at six acupoints on each side, always by the same operator. In G2 and G3, the laser device will be positioned over the same points as in G1, but will be switched off.

- Group 1 – experimental (*n* = 19) – low-level laser therapy over acupoints;
- Group 2 –positive control (*n* = 19) – occlusal splint usage;
- Group 3 – control (*n* = 19) – placebo laser therapy over acupoints;
- Group 4 – control (*n* = 19) – children without bruxism (Additional file 2)

The child will be seated comfortably in a room without noise or sound interference and positioned with the Frankfurt plane parallel to the floor. A total of twelve sessions of LLLT will be performed at a frequency of twice per week using a laser at wavelength of 786.94 nm with a conventional tip, energy density of 25 J/cm^2^, intensity of 1.675 mW/cm^2^, power of 70 mW, 1 J (J) per point for 20 s for a total of 12 J per session. Point application will be used in direct contact with the skin (spot area: 0.04 cm^2^) following the protocol suggested by Carvalho et al. [30] and Venezian et al. [21]. The Twin Flex Evolution® device (MM Optics) will be employed. A potentiometer will be used at the onset of the study to determine the effective mean power of the equipment and therapeutic doses applied during clinical use. The points irradiated are listed below with their respective explanations:

- IG-4 (Hegu) - has a direct and strong influence on the face, eyes, ears, nose and mouth. It is also used to calm the mind and relieve anxiety. This point is located in the patient’s hand at the base between the thumb and index finger
- F-3 (Taichong) - exerts a profound soothing effect on the mind. Its soothing action is increased when combined with IG-4.This point is located in the patient’s feet at the base between the hallux and index toe.
- VB-34 (Yanglingquan) - is an important point to relax the tendons whenever there are muscle contractions. This point is located in the upper distal part of gastrocnemius muscle.
- E-36 (Zusanli) - indicated to treat irritability, depression, insomnia, tiredness, fatigue and headache. This point is located below the patella in the lateral portion of the tibia.
- ID-19 (Tinggong) - indicated to treat problems in the ear region. This point is located in the anterior part of the tragus
- BP-6 (Sanyinjiao) - this is one of the most important points, with a broad scope of action. It has a strong soothing action on the mind and is generally used to treat insomnia. This point is located in the proximal end of the medial malleolus, the distal margin of the tibia (Additional files 3 and 4).

The patients in G3 (placebo LLLT) will receive the same treatment as those in G1, with the same equipment and a pen that emits a red guide light and a sound, but does not emit energy. After 30 days of follow up, the volunteers in this group will receive complementary treatment for the control of bruxism for ethical reasons.

For G2, the maxillary occlusal splint will be made with transparent acrylic resin and used on with upper arch with palatal and occlusal coverage. Impressions of the maxillary and mandibular arches will be made in alginate, poured in dental stone and mounted on a semi-adjustable articulator. The splint will be made in two layers of rose-colored wax and adapted to the maxillary teeth. The splint will have a thickness of 3 mm, contact with all teeth in centric relationship, distocclusion of the posterior teeth in laterality and protrusion, avoiding interferences on the swinging side with the canine guide extending from the vestibular to the lingual direction enough to prevent perforation and increase resistance to impact. An expander will also be installed so the splint will be able to accompany the growth dynamics. Following the protocol described by Hachmann et al., [20] the children will use the occlusal splint only at night for two months, with weekly adjustments of one quarter turn.

In G4 (control), children without bruxism will be evaluated. Therefore, this group will not be submitted to any bruxism treatment. This will be the control group.

### Study variables

All groups will be submitted to the evaluation of muscle activity using the BTS TMJOINT® electromyograph and the evaluation of bite force with a gnatodynamometer at baseline as well as one and six months after treatment. Salivary cortisol will be measured at baseline and after the therapeutic intervention.

### Protocol for electromyographic (EMG) evaluation of masticatory muscle and trapezius muscle

The electrical activity resulting from the activation of the masseter and temporal muscles as well as the descending fibres of the trapezius will be captured using a six-channel electromyograph (TMJOINT, BTS Engineering) with a bioelectric signal amplifier, wireless data transmission and disposable bipolar Ag/AgCl surface electrodes (Medical Trace®) measuring 10 mm in diameter. The EMG signal will be amplified with a 2000-fold gain and filtered within a frequency range of 20 to 450 Hz. Impedance of the equipment is >1015 Ω//0.2 pF and the common rejection mode ratio is 60/10 Hz 92 dB. The data will be captured and digitised at 1000 frames/s using the BTS MYOLA®52 software program. After cleaning the sites with 70% alcohol to diminish impedance between the skin and electrode [23], the self-adhesive surface electrodes will be attached over the belly of the muscle in the region with the most tonus (determined during moderate intercuspation) at a distance of 20 mm centre to centre, as suggested by the European Society Recommendations for Surface Eletromyography [23]. A reference electrode will be placed on the left wrist to impede the interference from outside noise.

The right and left masseter and anterior temporal muscles will be analysed under four conditions: i) at rest; ii) during maximum habitual intercuspation with a strip of Parafilm M® 40 between the molars for the collection of maximum voluntary contraction (MVC) of the muscles studied; iii) habitual chewing (isotonic contraction); and iv) maximum intercuspation (isometric contraction) without the Parafilm. Three readings will be made under each condition, with a two-minute interval between readings. The collection time will be 15 s for in the resting position, five seconds for MVC, 10 s for isotonic contraction and 10 s for isometric contraction [19, 31]. During the simulation of habitual chewing, a metronome set at 60 beats per minute will be used to standardise the process. The EMG signal captured during chewing will be rectified and normalised by the mean of the signal followed by the calculation of the root mean square (RMS) using a 500-ms moving window without overlap. The data will be normalised by the largest RMS obtained during MVC.

For the positioning of the electrodes on the descending fibres of the trapezius muscle, a point will be marked 2 cm lateral to the midpoint of the straight line between the posterior edge of the acromion and seventh cervical vertebra [19]. Prior to the readings, the participant will be instructed to sit in a chair with the shoulder and arm bare, back erect, knees flexed at 90° and feet apart for the collection of isometric MVC. Two non-elastic bands attached to each side of the base of the chair will be used to resist the movement of the raising of the shoulder during the reading. The volunteer will be instructed to raise the shoulders with maximum force for five seconds. The procedure will be repeated three times with a one-minute interval between readings. The highest value among the three readings will be used to normalise the EMG data of the descending fibres of the trapezius muscle. After a three-minute interval, the volunteer will be instructed to perform 90° abduction of both arms for 60 s, with the elbows completely extended and the forearms in pronation with the palms turned toward the floor. To monitor the position of the shoulder, two flexible rods positioned horizontally between the arms at a distance of 8 cm will be used to provide feedback of the tactile position [32].

Data processing of the EMG signals will be performed using specific routines developed in Matlab, version 7.1 (The MathWorks Inc., Natick, Massachusetts, USA).

### Protocol for analysis of bite force

Bite force will be measured using a digital dynamometer (DMD model, Kratos Equipamentos Industriais Ltda, Cotia, SP, Brazil) adapted for oral conditions. This device is an electronic bite force meter composed of a bite fork and module with a digital display connected by a wire. The readings will be made in the region of the first molars in Angle Class I [33] and free of caries. The volunteer will receive prior orientation and training to become familiar with the device. Six readings will be made – three on the right side and three on the left side, alternating sides between readings. Each reading will last five seconds and will be separated by a one-minute rest period. Bite force will be evaluated prior to the onset of treatment, at the end of treatment and 30 days after the end of treatment. The results will be computed and analysed statistically.

### Protocol for evaluation of occlusal contacts

Occlusal contacts will be determined during the electromyographic analysis following the method described by Ferrario et al. [34]. To evaluate symmetry of the temporal and masseter muscles, the overlap percentage coefficient will be computed, which is an index of the distribution of the symmetry of muscle activity ranging from 0% (asymmetry) to 100% (perfect symmetry). The torque coefficient will also be determined, which is the lateral displacement in contralateral activity of the masseter and temporal muscles ranging from 0% (absence of lateral displacement force) to 100% (maximum lateral displacement force) [27, 31]. The total mean activity of the masseter and temporal muscles will be analysed as area over time [27, 32–35]. A clinical examination will then be performed to determine the type of occlusion based on the Angle classification [33], which is the most practical, traditional system and is considered the gold standard in the literature. This system is based on anteroposterior relationships of the maxilla and mandible:

- Angle Class I (neutroclusion) – The mesiovestibular cusp of the permanent maxillary molar makes contact with the buccal groove of the permanent mandibular first molar;
- Angle Class II (distoclusion) – The mesial groove of the permanent mandibular first molar makes contact with the mesiobuccal cusp of the permanent maxillary first molar.
- Angel Class III (mesioclusion) – The mesial groove of the permanent mandibular first molar makes contact anterior to the mesiobuccal cusp of the permanent maxillary first molar.

### Protocol for evaluation of salivary cortisol

Saliva will be collected using a swab, which consists of a small ball of cotton on the end of a thin plastic tube. The swab will remain in the mouth for two minutes. The cotton will subsequently be placed in a centrifugation tube and stored at −20 °C until analysis [36]. Stress will be determined by the quantification of salivary cortical prior to the onset of treatment and on Day 50. For the study of circadian rhythm of cortisol, two saliva samples will be collected at the home of the participant after at least one hour of fasting and after oral hygiene with water: one at 9 am and one before sleep. The collections will be performed on a Sunday with orientation and using the recommendations of the manufacturer. The samples will be delivered the following day for analysis at Nove de Julho University.

### Protocol for evaluation of pain

The Wong-Baker FACES Pain Rating Scale will be used to evaluate pain. This is a self-reported scale that consists of a number of faces ranging from happy to crying. The scale will be explained to the children and they will indicate the face that best corresponds to their level of pain before and after treatment (Additional file 5).

### Statistical analyses

Descriptive statistics will be used first for the determination of point estimates. The Shapiro-Wilk test will be used to determine the normality of the data. The chi-square test, Student’s t-test and ANOVA will be employed for the analysis of associations between variables, with the level of significance set at 5% (*p* < 0.05). After the analysis of data distribution, ANOVA will be used for the evaluation of residuals and parametric tests will be used for the determination of pre-treatment conditions. Interval estimates will be used for the variables of interest to determine the estimates and perform comparisons. When necessary, transformation methods or non-parametric tests will be used in the data analysis.

## Discussion

The main objective of the proposed study is to compare the efficacy of low-level laser therapy administrated to acupoints and occlusal splint usage in children with bruxism. Obviously, it will not be possible to blind the participants submitted to occlusal splint usage (G2), as will occur with the other treatments (G1, G3 and G4), which can be considered a limitation of the study. However, this treatment cannot be excluded, since it is the gold standard in the literature, despite divergences of opinion [37].

Regarding the outcome variables, methods will be employed that can be reproducible in future studies and accessible to patients. Although polysomnography (sleep study) is the most effective for the diagnosis of bruxism, this method has limitations, such as the high cost and the amount of time required for its execution [38]. Moreover, this method had been criticised due to the fact that the patient is not in a familiar environment and children normally do not cooperate, which compromises the quality of the test [38].

The outcome variables muscle activity and bite force have been validated and used in previous studies [8, 39]. We expect an improvement in the distribution of the contact symmetry based on the chosen acupoints (regulation of anxiety/muscle relaxation). We do not expect the number of contacts to increase or intensify nor do we expect any improvement in the bruxism index (which will not be measured in this study). Some acupuncture points, especially IG-4 (Hegu), F-3 (Taichong) and BP-6 (Sanyinjiao), are indicated to alleviate anxiety and VB-34 (Yanglingquan) is used to treat muscle contraction.

The third outcome (level of salivary cortisol) has become increasingly common due to its non-invasive nature and the existence of accessible commercial kits [40–42]. We will act on some anxiety points, especially IG-4 (Hegu), F-3 (Taichong) and BP-6 (Sanyinjiao). Some authors [43, 44] state that benzodiazepines, especially clonazepam, are beneficial to adult patients with bruxism. However, the risk of dependency limits its use for prolonged periods. Moreover, such therapies are not indicated for children due to the side effects [45]. We expect treatment with acupoints to improve the anxiety of these children and cause a decrease of cortisol levels. While the attention given to the children during treatment could have a positive effect on decreasing anxiety, all groups will be subject to this bias.

To date, there are no randomised, controlled, clinical trials on this subject and the proposed study is expected to contribute to different fields of knowledge, such as pediatrics, dentistry, physical therapy, speech therapy, etc.

## Additional files

Additional file 1: Flow diagram. (PDF 76 kb)

Additional file 2: Chart 2 - Proposed experimental conditions. (DOCX 17 kb)

Additional file 3: Chart 1 - Points irradiated and respective explanations. (DOCX 12 kb)

Additional file 4: Figure S2. Low-level laser parameters. (DOCX 17 kb)

Additional file 5: Figure S1. Wong-Baker FACES Pain Rating Scale. (DOCX 37 kb)

## Abbreviations

EMG: Eletromyography
LLLT: Low level laser
MVC: Maximum voluntary contraction
