# Supplementary figures and images for: Evaluation of muscle activity, bite force and salivary cortisol in children with bruxism before and after low level laser applied to acupoints: study protocol for a randomised controlled trial
Source: BMC Complement Altern Med. 2017 Aug 8;17:391. doi: 10.1186/s12906-017-1905-y (PMC5549372; doi:10.1186/s12906-017-1905-y)

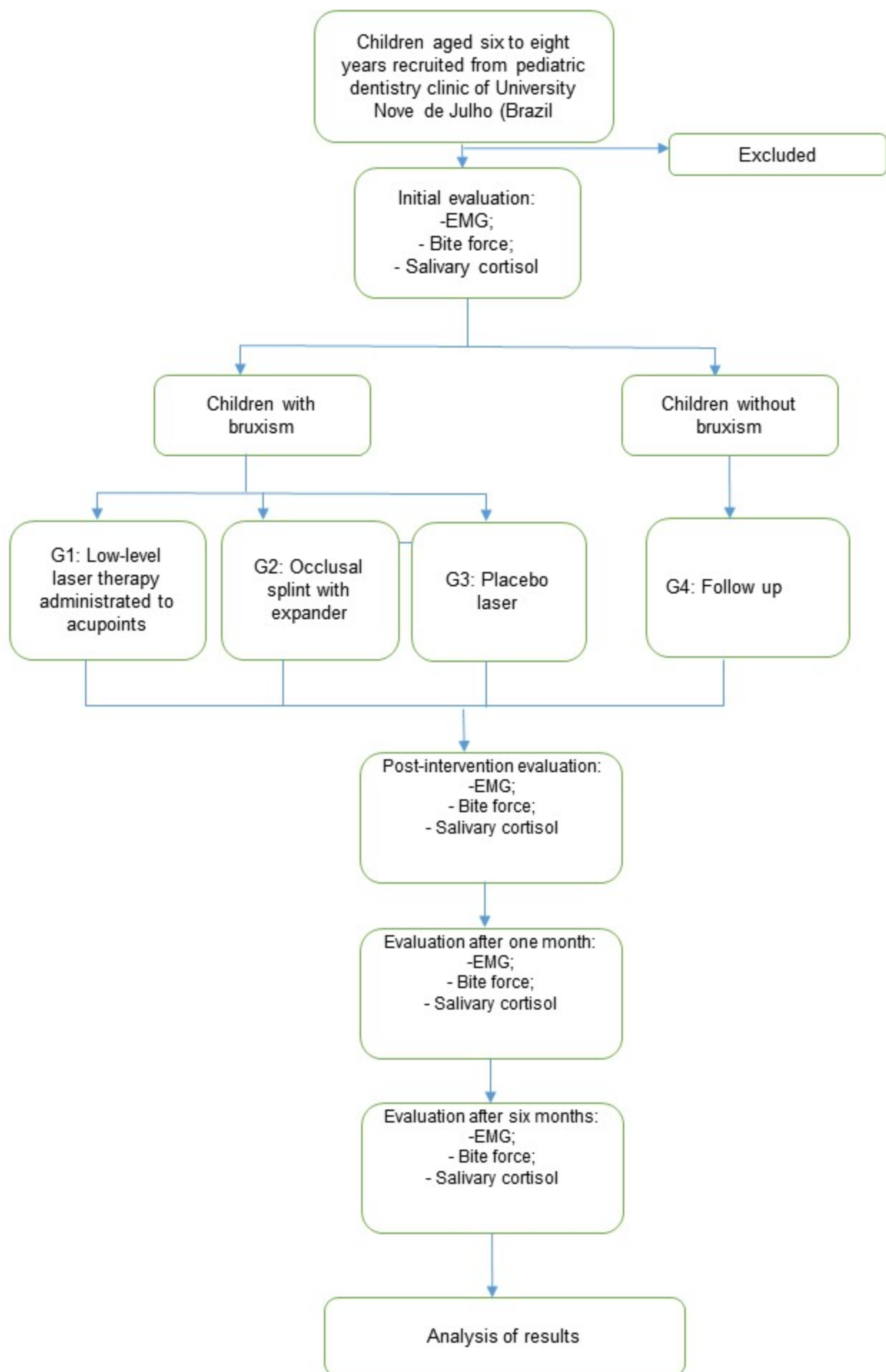

Supplement: Supplementary file 1 — Flow diagram. (PDF 76 kb) [file 12906_2017_1905_MOESM1_ESM.pdf]

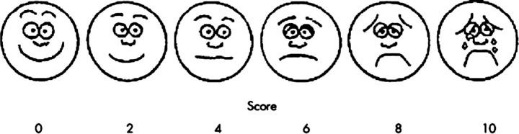


Figure 1. Wong Baker FACES Pain Rating Scale

Supplement: Supplementary file 5 — Wong-Baker FACES Pain Rating Scale. (DOCX 37 kb) [file 12906_2017_1905_MOESM5_ESM.docx]
