# Supplementary material for: Evaluation of muscle activity, bite force and salivary cortisol in children with bruxism before and after low level laser applied to acupoints: study protocol for a randomised controlled trial
Source: BMC Complement Altern Med. 2017 Aug 8;17:391. doi: 10.1186/s12906-017-1905-y (PMC5549372; doi:10.1186/s12906-017-1905-y)
Supplement: Supplementary file 2 — Chart 2 - Proposed experimental conditions. (DOCX 17 kb) [file 12906_2017_1905_MOESM2_ESM.docx]

| **Group** | **n** | **Therapeutic intervention** |
| --- | --- | --- |
| 1 | 19 | Low-level laser applied to acupoints |
| 2 | 19 | Occlusal splint with expander |
| 3 | 19 | Placebo laser |
| 4 | 19 | Control (without bruxism) |

Chart 2 - Proposed experimental conditions
