# Supplementary material for: Evaluation of muscle activity, bite force and salivary cortisol in children with bruxism before and after low level laser applied to acupoints: study protocol for a randomised controlled trial
Source: BMC Complement Altern Med. 2017 Aug 8;17:391. doi: 10.1186/s12906-017-1905-y (PMC5549372; doi:10.1186/s12906-017-1905-y)
Supplement: Supplementary file 3 — Chart 1 - Points irradiated and respective explanations. (DOCX 12 kb) [file 12906_2017_1905_MOESM3_ESM.docx]

| Acupoint | Description |
| --- | --- |
| IG-4 (Hegu) | This point has a strong, direct influence on the face, eyes, ears, nose and mouth. It is also used to calm the mind and relieve anxiety. |
| F-3 (Taichong) | This point has a profound calming effect on the mind. Its calming action is enhanced when combined with IG-4. |
| VB-34 (Yanglingquan) | This point in important to relaxing the tendons whenever muscles contract. |
| E-36(Zusanli) | This point is indicated for the treatment of irritability, depression, insomnia, weariness, fatigue and headache. |
| ID-19 (Tinggong) | This point is indicated for treating problems in the ear region. |
| BP-6 (Sanyinjiao) | This is one of the most important points, with a broad range of action. It is used to calm the mind and treat insomnia. |

Chart 1 - Points irradiated and respective explanations
