# Supplementary material for: Evaluation of muscle activity, bite force and salivary cortisol in children with bruxism before and after low level laser applied to acupoints: study protocol for a randomised controlled trial
Source: BMC Complement Altern Med. 2017 Aug 8;17:391. doi: 10.1186/s12906-017-1905-y (PMC5549372; doi:10.1186/s12906-017-1905-y)
Supplement: Supplementary file 4 — Low-level laser parameters. (DOCX 17 kb) [file 12906_2017_1905_MOESM4_ESM.docx]

|  | Infrared laser |
| --- | --- |
| Central wavelength (nm) | 786.94 |
| FWHM spectral width (nm) | 0.65 |
| Operation mode | Continuous |
| Mean maximum power (mW) | 70 |
| Polarization | Random |
| Aperture diameter (cm) | 0.226 |
| Radiance at aperture (mW/cm^2^) | 1.675 |
| Beam profile | Multimode |
| Beam area (cm^2^) | 0.04 |
| Radiance at target (mW/ cm^2^) | 1.675 |
| Exposure time (s) | 20 s per point |
| Radiant exposure (J/cm^2^) | 33.5 |
| Radiant energy (J) | 1 |
| Number of points irradiated | 12 |
| Irradiated area (cm^2^) | 0.48 |
| Administration mode | Contact |
| Number of sessions and frequency | 12 sessions (two per week) |
| Total irradiated energy (J) | 144 |

Figure 2 - Low-level laser parameters
